# Primaquine-induced Severe Hemolysis in the Absence of Concomitant Malaria: Effects on G6PD Activity and Renal Function

**DOI:** 10.4269/ajtmh.21-0834

**Published:** 2022-12-12

**Authors:** Nicholas M. Douglas, Kim A. Piera, Angela Rumaseb, Benedikt Ley, Nicholas M. Anstey, Ric N. Price

**Affiliations:** ^1^ Global and Tropical Health Division, Menzies School of Health Research and Charles Darwin University, Darwin, Australia;; ^2^ Department of Infectious Diseases, Christchurch Hospital, Canterbury District Health Board, Christchurch, New Zealand;; ^3^ Department of Medicine, University of Otago, Christchurch, New Zealand;; ^4^ Division of Infectious Diseases, Royal Darwin Hospital, Darwin, Australia;; ^5^ Centre for Tropical Medicine and Global Health, Nuffield Department of Clinical Medicine, University of Oxford, Oxford, United Kingdom;; ^6^ Mahidol Oxford Tropical Medicine Research Unit, Faculty of Tropical Medicine, Mahidol University, Bangkok, Thailand

## Abstract

Primaquine prevents relapses of *Plasmodium vivax* malaria but can cause severe hemolysis in patients with glucose-6-phosphate dehydrogenase (G6PD) deficiency. The clinical and laboratory features of this outcome are usually confounded by the clinical and hemolytic effects of concomitant malaria. We describe a case of severe hemolysis occurring after a total dose of 2.04 mg/kg of primaquine used for prophylaxis in a young, G6PD-deficient (Kaiping variant), Australian man without malaria. During acute hemolysis, he had markedly elevated urinary beta-2-microglobulin, suggestive of renal tubular injury (a well-recognized complication of primaquine-induced hemolysis). He also had albuminuria and significantly increased excretion of glycocalyx metabolites, suggestive of glomerular glycocalyx degradation and injury. We show that regularly dosed paracetamol given for its putative renoprotective effect is safe in the context of severe oxidative hemolysis. Acute drug-induced hemolysis transiently increases G6PD activity. Cases such as this improve our understanding of primaquine-induced hemolysis and ultimately will help facilitate widespread safe and effective use of this critically important drug.

A healthy 26-year-old man returned to Australia after 2 months’ military service in Peninsular Malaysia and was prescribed primaquine 15 mg twice daily for presumptive antirelapse therapy against *Plasmodium vivax* malaria. On the fourth day of primaquine therapy (eight doses taken [1.48 mg/kg]), he noticed his urine had become dark. He continued to take primaquine, but by the morning of the sixth day (11 doses taken [2.04 mg/kg]), he had developed generalized arthralgia, back pain, nausea, severe vomiting, dizziness, and dyspnea and therefore presented to hospital. He had not taken any medications other than primaquine before presentation and had not been febrile.

The patient was born to parents of Chinese ethnicity and had an unremarkable past medical history except for glucose-6-phosphate dehydrogenase (G6PD) deficiency, which had been diagnosed on routine military medical screening. He was a nonsmoker. On examination he was afebrile, pale, jaundiced, intermittently vomiting, and had a respiratory rate of 23 breaths per minute. There was no hepatosplenomegaly. His urine was dark brown in color with no visible sediment. Dipstick urinalysis revealed trace blood and no nitrites, protein or leukocytes (Figure 1), and urine microscopy revealed no significant bacterial growth, no cellular or proteinaceous casts, and normal red cell morphology.

**Figure 1.**
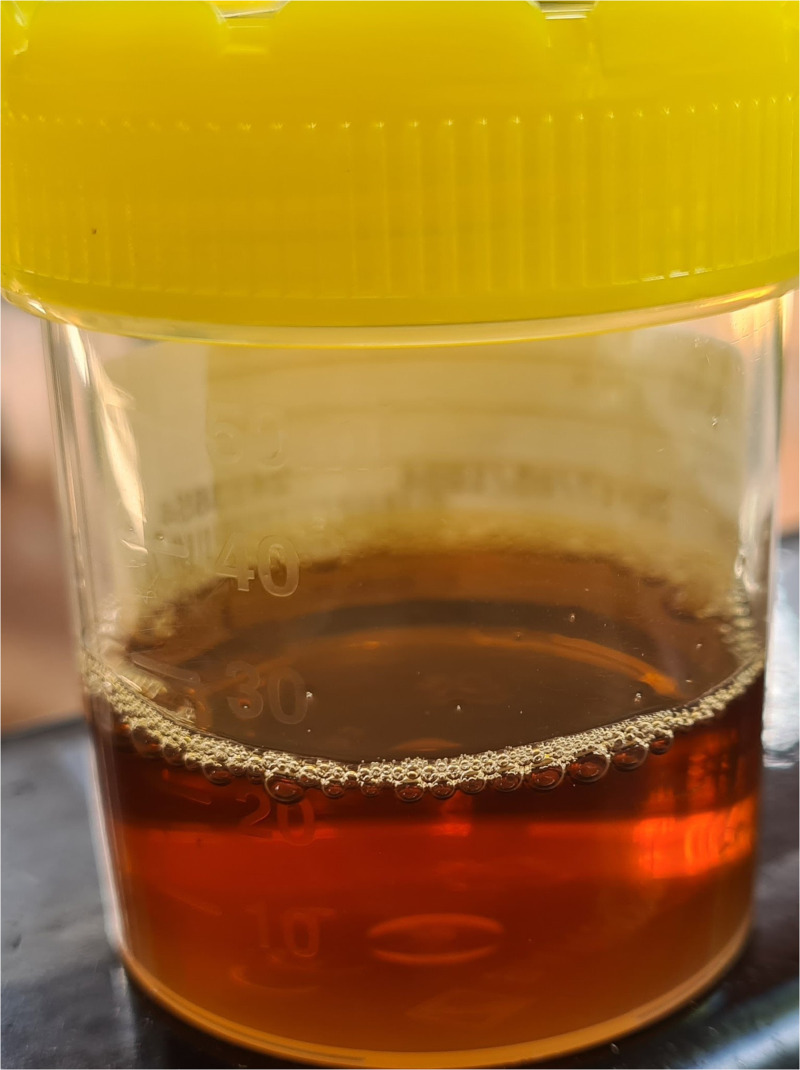
The patient’s urine on the day of presentation to hospital (sixth day of primaquine prophylaxis).

On the day of presentation, the patient’s hemoglobin was 88 g/L (reference range: 135–185 g/L), hematocrit 0.26 (0.4–0.54), and mean cell volume 96 fL (78–100 fL). Microcytic spherocytes, bite cells, and polychromasia were noted on blood film. Serial microscopy and rapid diagnostic tests for *P. falciparum* and common *Plasmodium* antigens were negative. Unconjugated bilirubin and lactate dehydrogenase were elevated (97 µmol/L [0–19 µmol/L] and 781 U/L [120–250 U/L], respectively), and haptoglobin was subnormal (<0.10 g/L [0.36–1.95 g/L]). A direct antiglobulin test was negative. Spectrophotometry confirmed deficiency of the glucose-6-phosphate dehydrogenase enzyme (2.2 U/g Hb; reference range: 5.0–13.0 U/g Hb), and hemoglobin electrophoresis did not show any concomitant hemoglobinopathies. Plasma cell-free hemoglobin concentration was elevated (120,004 ng/mL [< 50,000 ng/mL]). He had albuminuria (urinary albumin-to-creatinine ratio 7.4 [< 2.5]), increased urinary glycocalyx metabolite excretion (11.16 total glycosaminoglycans/mol creatinine)^1^ and markedly elevated urinary beta-2-microglobulin (40,019 µg/L; normal range < 300). Plasma creatinine and urea were normal (66 µmol/L [60–110 µmol/L] and 7.0 mmol/L [3.0–7.5 mmol/L], respectively).

A diagnosis of severe, primaquine-induced hemolysis was made. The patient’s primaquine prophylaxis was discontinued, and he was prescribed antiemesis with ondansetron and paracetamol 1 g four times daily for analgesia and renal protection (after blood and urine collection).^2^ He was admitted to the ward for further monitoring. Despite primaquine cessation, the next day, his hemoglobin decreased to 67 g/L, although falling lactate dehydrogenase and bilirubin concentrations suggested less active hemolysis (Figure 2). He did not require a blood transfusion. Within 48 hours, the hemoglobin had risen and the cell free hemoglobin, urine albumin:creatinine ratio, urinary glycocalyx metabolite excretion, and urinary beta-2 microglobulin (15 µg/L) had all normalized (Figure 2). The patient’s nausea and back pain had also settled, and his urine had returned to a normal color. Paracetamol was ceased on day 5. The alanine transaminase concentration peaked at 83 U/L (< 50 U/L) on day 5 (paracetamol concentration < 10 mg/L), and gradually normalized thereafter.

**Figure 2.**
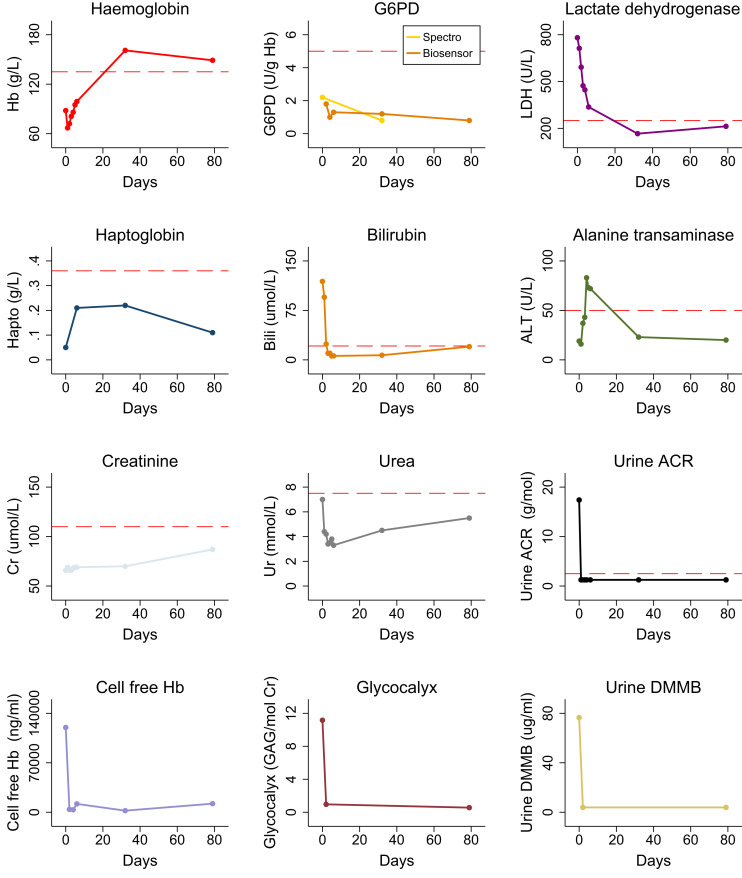
Trajectory of laboratory parameters after diagnosis of severe primaquine-induced oxidative hemolysis. ACR = albumin:creatinine ratio; ALT = alanine transaminase; Bili = bilirubin; Cr = creatinine; G6PD = glucose-6-phosphate dehydrogenase; GAG = total glycosaminoglycans; Hapto = haptoglobin; Hb = hemoglobin; LDH = lactate dehydrogenase; Ur = urea; Urine DMMB = urine 1,9-dimethylmethylene blue assay (measures urinary glycosaminoglycans).

The patient was discharged on day 6 and subsequently followed in the outpatient setting for another 3 months. At 1 month, he reported persistent mild exertional dyspnea, despite a hemoglobin concentration of 161 g/dL, but this had resolved by 3 months. Repeat G6PD activity by spectrophotometry at 1 month and 3 months was 0.8 U/g Hb and 0.7 U/g Hb, respectively. Gene sequencing showed that the patient carried the Kaiping G6PD variant (1388 G>A), which is highly prevalent across East Asia and typically results in G6PD activity < 10% of normal.^3^ The patient’s renal and hepatic function remained normal throughout follow-up, and his blood film returned to normal.

Primaquine, an 8-aminoquinoline, is the only widely available drug that kills the liver stages (hypnozoites) of *Plasmodium vivax* and *P. ovale* and thus prevents future relapses.^4^ Although primaquine radical cure of both the blood and liver stages of the parasite is important for reducing transmission of *P. vivax,* its widespread use is limited because it can cause severe hemolysis in patients with G6PD deficiency, an enzymopathy that is most prevalent in malaria-endemic areas.^5^ Assessment of the frequency and clinical features of severe primaquine-induced hemolysis in endemic regions has been confounded by the difficulty of disentangling the hemolytic effects of concomitant malaria.^6^^–^^8^ Because most serious hemolytic events occur in resource-poor settings where extensive laboratory investigation is not feasible, the current case provided an important opportunity to investigate comprehensively the natural history, laboratory features, and deleterious effects of primaquine-induced hemolysis in the absence of clinical malaria.

In normal individuals, total intraerythrocytic G6PD capacity well exceeds requirements to reduce oxidative free radicals generated by the active metabolites of primaquine. Patients with inherited G6PD deficiency may have insufficient total G6PD activity to prevent primaquine-induced hemolysis with older erythrocytes being the most susceptible due to a physiological reduction in antioxidant capacity with age.^9^ Erythropoiesis increases in response to accelerated loss of senescent erythrocytes, and combined, these factors result in reduced mean erythrocyte age and a transient increase in total G6PD activity.^10^ The patient described in this report had a G6PD activity of 2.2U/g Hb at presentation, equivalent to approximately 30% of the adult male mean, the threshold used to differentiate intermediate from severe deficiency and guide the use of primaquine. After 3 months, his G6PD activity had fallen to 0.7 U/g Hb (approximately 9.5% activity), placing him in the severely deficient category, consistent with the Kaiping variant. All causes of hemolysis, including peripheral *Plasmodium* parasitemia, have the potential to increase measured G6PD activity through one or both aforementioned mechanisms. This case demonstrates the dynamic nature of G6PD activity and suggests that vulnerability to drug-induced hemolysis may also fluctuate.^11^

Although blood creatinine and urea (both relatively crude measures of renal function) remained normal throughout our patient’s hospitalization and subsequent follow-up, the markedly elevated beta-2 microglobulin excretion suggested tubular and glomerular injury,^12^ in keeping with well-established renal tubular injury arising from primaquine-induced hemolysis.^8^ His degree of albuminuria on presentation (urine albumin-to-creatinine ratio 17.4) suggested increased permeability of the glomerular filtration barrier. The high urinary excretion of glycocalyx metabolites, at concentrations comparable to the highest levels seen in severe malaria,^1^^,^^13^ suggested significant glomerular glycocalyx degradation.^14^^,^^15^ The importance of glomerular glycocalyx loss in glomerular permeability and acute kidney injury is increasingly recognized^15^ but cannot be visualized on histopathology, such as that used in previous reports of primaquine hemolysis.^8^ An elevated admission plasma osteoprotegerin (Figure 2) highlights systemic hemolysis-induced endothelial activation,^16^ but plasma syndecan-1 levels were normal throughout, suggesting lack of significant systemic glycocalyx degradation.^1^ Both severe and nonsevere falciparum and knowlesi malaria cause glomerular glycocalyx degradation,^1^^,^^13^ but it is not known to what extent this is attributable to renal microvascular parasite sequestration or concomitant hemolysis. Our patient did not have concomitant malaria, and thus his case suggests that as well as causing renal tubular injury, primaquine-induced hemolysis alone may also cause glomerular glycocalyx degradation and injury. These have been proposed as major mechanisms of acute kidney injury in post-artesunate delayed hemolysis.^17^ This also supports the notion that oxidative damage to renal tubules and glomerular glycocalyx from cell-free heme released during hemolysis are significant contributors to acute kidney injury in malaria.^2^^,^^16^^,^^18^ Our data support the evidence in other settings that tubular epithelial cell injury may not be the only mechanism of hemolysis-induced renal damage.^19^

G6PD is an important antioxidant enzyme in all cells, but renal tissue has less constitutive G6PD than other tissues.^20^ In G6PD-deficient individuals, renal cells will have even less G6PD-mediated antioxidant defense. Although it is likely that most of the primaquine-induced oxidative nephrotoxicity in G6PD-deficiency is mediated by heme, we speculate that there may be an additional component of direct primaquine-induced oxidant damage in G6PD-deficient glomerular and tubular cells, independent of heme. Even mild acute kidney injury can increase the risk of chronic kidney disease and associated morbidities, and therefore it should be prevented as a priority.^21^^,^^22^

Regular paracetamol has been shown to have renoprotective effects in severe falciparum and knowlesi malaria, and this is particularly apparent in patients with prominent hemolysis.^2^^,^^23^ It is postulated that paracetamol-induced reduction of toxic ferryl heme to its ferric state quenches heme-ferryl radicals.^24^ Our patient received paracetamol 1 g four times daily for 5 days, both for analgesia of his severe back pain and its putative renoprotective effect in hemolysis.^2^ The modest rise in alanine transaminase observed was comparable to that seen in patients with malaria given regularly dosed paracetamol as part of clinical trials.^2^^,^^23^ None of these trial patients, or our patient, met Hy’s law of hepatotoxicity. It is uncertain whether paracetamol contributed to the rapid resolution of hemolysis-induced kidney injury in our patient, but our findings suggest that regularly dosed paracetamol in drug-induced hemolysis is safe in individuals with severe G6PD-deficiency.

Our patient was prescribed primaquine even though he had a known history of G6PD deficiency and had not been in a malaria-endemic region of Malaysia. This likely reflects lack of familiarity with use of primaquine in our nonendemic setting and provides a salutary reminder to ensure that both indications and contraindications are reviewed before prescribing infrequently used drugs.

In summary, our case of primaquine-induced hemolysis in an aparasitemic patient with severe G6PD deficiency highlights that 1) hemolysis transiently increases baseline G6PD activity, 2) regular paracetamol can be given safely in G6PD-deficient patients with hemolysis, and 3) severe primaquine-induced hemolysis alone may cause not only tubular injury but likely also glomerular glycocalyx degradation and injury, presumably due to oxidative damage from cell free heme. Safer and more effective primaquine regimens for patients with vivax malaria are needed if global elimination targets for *P. vivax* are to be achieved. A greater understanding of the natural history and pathogenetic mechanisms of primaquine-induced hemolysis and kidney injury will facilitate widespread use of this important drug.
